# Exercise electrocardiography stress testing in suspected angina and non-obstructive coronary arteries

**DOI:** 10.1093/eurheartj/ehaf208

**Published:** 2025-05-21

**Authors:** Robert Sykes, Andrew J Morrow, Bethany Stanley, Daniel Ang, Giles Roditi, David Stobo, David Corcoran, Ninian N Lang, Ahmed Mahrous, Robin Young, David Carrick, Ross McGeoch, Margaret B McEntegart, Alex McConnachie, Novalia P Sidik, Colin Berry

**Affiliations:** West of Scotland Heart and Lung Centre, NHS Golden Jubilee National Hospital, Glasgow G81 4DY, UK; British Heart Foundation Glasgow Cardiovascular Research Centre, School of Cardiovascular and Metabolic Health, University of Glasgow, 126 University Place, Glasgow G12 8TA, UK; West of Scotland Heart and Lung Centre, NHS Golden Jubilee National Hospital, Glasgow G81 4DY, UK; British Heart Foundation Glasgow Cardiovascular Research Centre, School of Cardiovascular and Metabolic Health, University of Glasgow, 126 University Place, Glasgow G12 8TA, UK; Robertson Centre for Biostatistics, School of Health and Wellbeing, University of Glasgow, Glasgow, UK; West of Scotland Heart and Lung Centre, NHS Golden Jubilee National Hospital, Glasgow G81 4DY, UK; British Heart Foundation Glasgow Cardiovascular Research Centre, School of Cardiovascular and Metabolic Health, University of Glasgow, 126 University Place, Glasgow G12 8TA, UK; Department of Radiology, Glasgow Royal Infirmary, NHS Greater Glasgow and Clyde Health Board, Glasgow, UK; Department of Radiology, Queen Elizabeth University Hospital, NHS Greater Glasgow and Clyde Health Board, Glasgow, UK; Department of Cardiology, Queen Elizabeth University Hospital, NHS Greater Glasgow and Clyde Health Board, Glasgow, UK; British Heart Foundation Glasgow Cardiovascular Research Centre, School of Cardiovascular and Metabolic Health, University of Glasgow, 126 University Place, Glasgow G12 8TA, UK; Department of Cardiology, Queen Elizabeth University Hospital, NHS Greater Glasgow and Clyde Health Board, Glasgow, UK; Department of Cardiology, Raigmore Hospital, Inverness, UK; Robertson Centre for Biostatistics, School of Health and Wellbeing, University of Glasgow, Glasgow, UK; Department of Cardiology, University Hospital Hairmyres, East Kilbride, UK; Department of Cardiology, University Hospital Hairmyres, East Kilbride, UK; West of Scotland Heart and Lung Centre, NHS Golden Jubilee National Hospital, Glasgow G81 4DY, UK; British Heart Foundation Glasgow Cardiovascular Research Centre, School of Cardiovascular and Metabolic Health, University of Glasgow, 126 University Place, Glasgow G12 8TA, UK; Department of Cardiology, Columbia University Medical Center, New York, NY, USA; Robertson Centre for Biostatistics, School of Health and Wellbeing, University of Glasgow, Glasgow, UK; West of Scotland Heart and Lung Centre, NHS Golden Jubilee National Hospital, Glasgow G81 4DY, UK; British Heart Foundation Glasgow Cardiovascular Research Centre, School of Cardiovascular and Metabolic Health, University of Glasgow, 126 University Place, Glasgow G12 8TA, UK; West of Scotland Heart and Lung Centre, NHS Golden Jubilee National Hospital, Glasgow G81 4DY, UK; British Heart Foundation Glasgow Cardiovascular Research Centre, School of Cardiovascular and Metabolic Health, University of Glasgow, 126 University Place, Glasgow G12 8TA, UK

**Keywords:** Exercise stress testing, Coronary computed tomography angiography, Angina with no obstructive coronary arteries, Microvascular angina, Vasospastic angina

## Introduction

In clinical guidelines, exercise electrocardiography stress testing (EST) is recommended for risk stratification in patients with angina, whereas coronary computed tomography angiography (CCTA) is recommended for the diagnosis of obstructive coronary artery disease.^1^ The exercise EST may be considered (class of recommendation IIb) as an alternative option when non-invasive imaging tests are unavailable. There is no recommendation on the use of exercise EST following CCTA.^1^ This gap in evidence is relevant since most individuals with stable angina who undergo CCTA do not have obstructive coronary artery disease^2^ and angina/ischaemia with non-obstructive coronary arteries (ANOCA/INOCA) may be causally relevant.^1^ A strategy of CCTA coupled with exercise EST may be diagnostically useful in suspected ANOCA/INOCA, but prior studies have involved a retrospective design^3,4^ and selected populations with a limited sample size.^5–7^

The aim of this study was to assess the diagnostic accuracy of exercise EST for identifying endotypes (microvascular angina, vasospastic angina, or both) in a community-dwelling population with suspected ANOCA/INOCA according to invasive functional testing [coronary flow reserve (CFR), index of microvascular resistance (IMR), and acetylcholine vasomotor responses] as a reference. We hypothesized that ischaemic group status by exercise EST criteria has at least moderate sensitivity and specificity for coronary endotypes, supporting adoption of the exercise EST in this population.

## Methods

The CorCTA trial involved 250 outpatients with suspected ANOCA/INOCA post-CCTA who underwent invasive endotyping for microvascular angina and/or vasospastic angina or non-cardiac chest pain.^8^ This study describes a predefined secondary analysis, and the methods have been described.^8^ Eligible patients provided informed consent and then completed the Rose Angina^9^ and Seattle Angina Questionnaires (SAQ).^10^

The exercise EST involved the Bruce treadmill protocol, with myocardial ischaemia defined as ≥.1 mV ST-segment depression 60 ms from the J-point (ischaemic group).

Participants with suspected ANOCA/INOCA underwent functional coronary angiography involving guide-wire–based coronary thermodilution at rest and during adenosine hyperaemia (Abbott, Irvine, CA, USA; Coroventis, Uppsala/Sweden) followed by intracoronary infusion of acetylcholine (.182 μg/mL, 1.82 μg/mL, 18.2 μg/mL; 2 mL/min for 2 min).

### Statistics

The analysis population was restricted to patients who performed an exercise EST and for those where ischaemic group status (ST-segment deviation) was definable. Categorical outcomes were compared between groups (ischaemic and non-ischaemic groups; final diagnosis endotype groups) using Fisher’s exact test, and continuous outcomes were compared between groups using the Wilcoxon–Mann–Whitney test (for data with a skewed distribution) and Student’s *t*-test (for normal distributed data). A two-tailed analysis was performed, and a *P* < .05 was considered as statistically significant.

Measures of diagnostic accuracy (with 95% confidence intervals) of ischaemic group status for coronary microvascular dysfunction subgroups were assessed.

## Results

### Population

Overall, 163 individuals [mean age, 54.6 years, body mass index 30.9 kg/m^2^, 102 (62.6%) women] with symptoms of suspected angina and ischaemic [*n* = 45 (27.6%)] or non-ischaemic [*n* = 118 (72.4%)] group status defined by ST-segment deviation on exercise EST were included.

### Symptoms and ischaemia status by exercise electrocardiography stress testing

Of the 163 participants assessed using the Rose Angina Questionnaire, 91 (55.8%) had definite angina and 72 (44.2%) had probable angina. Compared with the non-ischaemic group, in the ischaemic group, angina stability measured by the SAQ was lower (worse) [48.3 (16.3) vs. 55.2 (22.2); *P* = .034].

### Non-invasive coronary computed tomography angiography findings

Of the 163 participants, 68 (41.7%) had no evidence of coronary atherosclerosis [ischaemic (*n* = 15, 33.3%) vs. non-ischaemic groups (*n* = 53, 44.9%); *P* = .215].

Myocardial bridging was most prevalent in patients with vasospastic angina (29%) compared with other groups (normal coronary function 2.1%, microvascular angina 4.7%, mixed microvascular angina and vasospastic angina 14.3%; *P* = .002). Myocardial bridging did not occur in the ischaemic group [0 vs. 13 (11.0%); *P* = .020].

### Angina/ischaemia with non-obstructive coronary arteries endotypes

Eighty-three (50.9%) participants had microvascular angina, 20 (12.3%) had vasospastic angina, 14 (8.6%) had both, and 46 (28.2%) had no abnormality, and the distribution of these endotypes was similar in the ischaemic and non-ischaemic groups (*P* = .907). The IMR, CFR, microvascular spasm, and epicardial vasospasm in the left anterior descending coronary artery were not different between groups. Thirteen (28.9%) of the 45 patients in the ischaemic group had normal coronary function, indicating a probable false positive exercise EST result and no sex association (3 of 12 males vs. 10 of 33 females; *P* = 1.000).

### Specificity and sensitivity

Ischaemic group status had moderate specificity (72.5%) to detect microvascular dysfunction (CFR < 2.5 and IMR ≥ 25 and microvascular spasm by acetylcholine) but low sensitivity (30.0%) (*Table 1*). The negative predictive value for the exercise EST was high for adenosine-CFR < 2.5, adenosine-CFR < 2.5, and microvascular spasm (acetylcholine response) and the combination of adenosine-CFR < 2.5 or microvascular spasm (acetylcholine response) or adenosine-IMR ≥ 25 (.814, .924, and .941, respectively).

**Table 1:** The exercise EST in ANOCA, ischaemic group status and endotypes of coronary microvascular dysfunction

(A) The exercise EST at baseline summarized for the study population overall and by ischaemic group status (ST-segment deviation) in 163 individuals					
		All (*N* = 163)	Ischaemic group (*N* = 45)	Non-ischaemic group (*N* = 118)	*P*-value
Symptoms elicited during testing					
Limiting angina	*N* (*N*_missing_) *N* (%) yes	163 (0) 30 (18.4%)	45 (0) 7 (15.6%)	118 (0) 23 (19.5%)	.655
Non-limiting angina	*N* (*N*_missing_) *N* (%) yes	163 (0) 53 (32.5%)	45 (0) 18 (40.0%)	118 (0) 35 (29.7%)	.262
Breathlessness	*N* (*N*_missing_) *N* (%) yes	163 (0) 59 (36.2%)	45 (0) 20 (44.4%)	118 (0) 39 (33.1%)	.204
Fatigue	*N* (*N*_missing_) *N* (%) yes	163 (0) 37 (22.7%)	45 (0) 6 (13.3%)	118 (0) 31 (26.3%)	.095
Exercise duration (minutes)	*N* (*N*_missing_) Mean (SD) Median (IQR) [Min, max]	163 (0) 7.1 (2.5) 7.2 (5.7, 9.0) [.0, 13.0]	45 (0) 7.0 (2.3) 6.8 (5.7, 9.0) [2.2, 13.0]	118 (0) 7.1 (2.5) 7.2 (5.7, 9.1) [.0, 13.0]	.468
Metabolic equivalents	*N* (*N*_missing_) Mean (SD) Median (IQR) [Min, max]	149 (14) 9.6 (8.3) 9.3 (7.0, 10.2) [2.2, 101.0]	44 (1) 10.6 (14.1) 8.0 (7.0, 10.1) [4.6, 101.0]	105 (13) 9.2 (3.8) 10.1 (7.0, 10.4) [2.2, 38.7]	.186
Duke treadmill score	*N* (N_missing_) Mean (SD) Median (IQR) [Min, max]	163 (0) 3.2 (4.7) 3.6 (.0, 6.6) [−9.6, 12.0]	45 (0) .3 (4.3) 1.5 (−2.4, 3.5) [−9.6, 10.5]	118 (0) 4.4 (4.3) 5.2 (1.8, 8.0) [−6.8, 12.0]	***P* < .001**
Duke treadmill score using metabolic equivalents	*N* (*N*_missing_) Mean (SD) Median (IQR) [Min, max]	163 (0) 5.3 (10.2) 4.7 (.0, 8.2) [−8.0, 94.5]	45 (0) 4.9 (16.9) 3.1 (−.7, 4.7) [−7.5, 94.5]	118 (0) 5.4 (6.1) 6.4 (1.7, 10.0) [−8.0, 38.7]	***P* < .001**

(B) Diagnostic accuracy (with 95% CI) of ischaemic group status during the exercise EST to detect coronary microvascular dysfunction in 163 individuals							
Measure	Group 1 (CFR < 2.5) (*N* = 33)	Group 2 (MV spasm) (*N* = 70)	Group 3 (IMR ≥ 25) (*N* = 56)	Group 4 (CFR < 2.5 and MV spasm) (*N* = 15)	Group 5 (CFR < 2.5 and IMR ≥ 25) (*N* = 22)	Group 6 (MV spasm and IMR ≥ 25) (*N* = 31)	Group 7 (CFR < 2.5, MV spasm, and IMR ≥ 25) (*N* = 10)
Sensitivity (95% CI)	33.3% (18.0%, 51.8%)	30.0% (19.6%, 42.1%)	21.4% (11.6%, 34.4%)	40.0% (16.3%, 67.7%)	22.7% (7.8%, 45.4%)	19.4% (7.5%, 37.5%)	30.0% (6.7%, 65.2%)
Specificity (95% CI)	73.8% (65.4%, 81.2%)	74.2% (64.1%, 82.7%)	69.2% (59.5%, 77.7%)	73.6% (65.8%, 80.5%)	71.6% (63.4%, 78.9%)	70.5% (61.9%, 78.1%)	72.7% (65.0%, 79.6%)
PPV (95% CI)	24.4% (12.9%, 39.5%)	46.7% (31.7%, 62.1%)	26.7% (14.6%, 41.9%)	13.3% (5.1%, 26.8%)	11.1% (3.7%, 24.1%)	13.3% (5.1%, 26.8%)	6.7% (1.4%, 18.3%)
NPV (95% CI)	81.4% (73.1%, 87.9%)	58.5% (49.0%, 67.5%)	62.7% (53.3%, 71.4%)	92.4% (86.0%, 96.5%)	85.6% (77.9%, 91.4%)	78.8% (70.3%, 85.8%)	94.1% (88.3%, 97.6%)

The bold highlight visually conveys that these two results are statistically significant (p-value), whereas the other values are not.

The study population is restricted to patients that performed an exercise EST and for those where ischaemic group status was able to be defined. Between-group comparisons assessed using Student’s *t*-test (normal distributed data) or the Mann–Whitney *U* test (skewed data) for continuous variables and the Fisher’s exact test for categorical variables.

CFR, coronary flow reserve; CI, confidence interval; EST, electrocardiography stress test; IMR, index of microvascular resistance; MV, microvascular; NPV, negative predictive value; PPV, positive predictive value.

## Discussion

The main findings were, first, 27.6% of individuals with suspected ANOCA/INOCA fulfilled the exercise EST criteria for myocardial ischaemia, which is expectedly lower compared with populations downstream in the care pathway who have been selected for invasive management^7^; second, ischaemic group status was associated with worse angina stability (SAQ); third, ischaemic group status had high negative predictive value for coronary microvascular dysfunction.

This novel design included prospective enrolment in multiple centres, minimal selection criteria, validated patient-reported outcome measures, central review of the exercise EST, a reference invasive centre, comprehensive invasive endotyping, and an independent clinical trials unit.^7^ Second, the population was larger than in prior studies^5–7^ and included patients with undifferentiated chest pain upstream in the outpatient setting, this being distinct from prior studies which included patients downstream in the care pathway following selection for invasive management.^6^ Finally, the exercise EST involved the Bruce protocol in outpatient clinics, implying the results are transferable to daily practice. The exercise EST is widely available, brief, inexpensive, repeatable, and free of ionizing radiation (noting most individuals with ANOCA/INOCA are female). Our study brings together non-invasive exercise EST and invasive endotyping, addressing an evidence gap on ANOCA/INOCA.^1^

We propose a clinical algorithm: in patients with suspected angina, CCTA should be considered initially.^1^ Then, in patients with suspected ANOCA/INOCA post-CCTA, the exercise EST represents a rule-out strategy for microvascular endotypes, exercise capacity, and prognosis. Alternative non-invasive stress test modalities are also useful.^1,3^

### Limitations

Non-invasive ischaemia testing with other diagnostic modalities was not included. No adjustment has been made for multiple testing.

### Data sharing

The anonymized data are available upon reasonable request.

## Declarations

### Data Availability

Anonymized data may be available upon reasonable request.

### Pre-registered Clinical Trial Number

Clinicaltrials.gov Registration: NCT03477890.

## References

[1] Vrints C, Andreotti F, Koskinas KC, Rossello X, Adamo M, Ainslie J, et al 2024 ESC guidelines for the management of chronic coronary syndromes. Eur Heart J 2024;45:3415–537. 10.1093/eurheartj/ehae177 39210710

[2] SCOT-HEART investigators . CT coronary angiography in patients with suspected angina due to coronary heart disease (SCOT-HEART): an open-label, parallel-group, multicentre trial. Lancet 2015;385:2383–91. 10.1016/S0140-6736(15)60291-4 25788230

[3] Pargaonkar VS, Kobayashi Y, Kimura T, Schnittger I, Chow EKH, Froelicher VF, et al Accuracy of non-invasive stress testing in women and men with angina in the absence of obstructive coronary artery disease. Int J Cardiol 2019;282:7–15. 10.1016/j.ijcard.2018.10.073 30527992 PMC9017854

[4] Lopez DM, Divakaran S, Gupta A, Bajaj NS, Osborne MT, Zhou W, et al Role of exercise treadmill testing in the assessment of coronary microvascular disease. JACC Cardiovasc Imaging 2022;15:312–21. 10.1016/j.jcmg.2021.07.013 34419395 PMC8831663

[5] Vandeloo B, Andreini D, Brouwers S, Mizukami T, Monizzi G, Lochy S, et al Diagnostic performance of exercise stress tests for detection of epicardial and microvascular coronary artery disease: the UZ clear study. EuroIntervention 2023;18:e1090–8. 10.4244/EIJ-D-22-00270 36147027 PMC9909457

[6] Sinha A, Dutta U, Demir OM, De Silva K, Ellis H, Belford S, et al Rethinking false positive exercise electrocardiographic stress tests by assessing coronary microvascular function. J Am Coll Cardiol 2024;83:291–9. 10.1016/j.jacc.2023.10.034 38199706 PMC10790243

[7] Ford TJ, Yii E, Sidik N, Good R, Rocchiccioli P, McEntegart M, et al Ischemia and no obstructive coronary artery disease: prevalence and correlates of coronary vasomotion disorders. Circ Cardiovasc Interv 2019;12:e008126. 10.1161/CIRCINTERVENTIONS.119.008126 31833416 PMC6924940

[8] Sidik NP, Stanley B, Sykes R, Morrow AJ, Bradley CP, McDermott M, et al Invasive endotyping in patients with angina and no obstructive coronary artery disease: a randomized controlled trial. Circulation 2024;149:7–23. 10.1161/CIRCULATIONAHA.123.064751 37795617

[9] Rose G, McCartney P, Reid DD. Self-administration of a questionnaire on chest pain and intermittent claudication. Br J Prev Soc Med 1977;31:42–8. 10.1136/jech.31.1.42 856370 PMC478990

[10] Thomas M, Jones PG, Arnold SV, Spertus JA. Interpretation of the Seattle angina questionnaire as an outcome measure in clinical trials and clinical care: a review. JAMA Cardiol 2021;6:593–9. 10.1001/jamacardio.2020.7478 33566062 PMC8651216

